# Oral Health in Physical Therapy Curriculum: An Essential Component for the Practice of Physical Therapy

**DOI:** 10.1093/geroni/igaf122.2952

**Published:** 2025-12-31

**Authors:** Julie Hartmann, Tara Granada, Victoria Steele, Zachary Tussey

**Affiliations:** Bellarmine University, Louisville, Kentucky, United States; Bellarmine University, Louisville, Kentucky, United States; Bellarmine University, Louisville, Kentucky, United States; Bellarmine University, Louisville, Kentucky, United States

## Abstract

Literature has revealed a link between oral health and overall systemic health and that a correlation between poor oral health and several chronic diseases, such as diabetes and heart disease exist. Encountering patients with comorbidities that are associated with poor oral health are of high frequency in the practice of physical therapy. It is imperative that physical therapists are equipped with tools necessary to screen for poor oral health and appropriately refer to a dental health professional. The physical therapist should have the knowledge and ability to perform oral health screening as indicated for an older individual in order to provide a comprehensive examination. This reinforces the importance of incorporating oral health content and interprofessional collaboration with dentistry into Doctor of Physical Therapy (DPT) curriculum. Suggestions have been provided for integration into a sample University’s DPT curriculum, which have been categorized by body systems, disease processes with delineation of the associated oral health connection. The purpose of this work is threefold. First, it is pertinent to relay the importance that a healthcare professionals’ recognition of integrating oral health screening into their practice may elevate the standard of care provided to older adults. Second, a framework has been provided to encourage the incorporation of oral health screening and education into physical therapy practice for older adults. Lastly, it would be beneficial to integrate oral health information into physical therapy curriculum to improve educational opportunities for physical therapy students as well as to promote collaboration with dentistry.

